# Interactive Tree of Life (iTOL) v6: recent updates to the phylogenetic tree display and annotation tool

**DOI:** 10.1093/nar/gkae268

**Published:** 2024-04-13

**Authors:** Ivica Letunic, Peer Bork

**Affiliations:** biobyte solutions GmbH, Bothestr 142, 69126 Heidelberg, Germany; EMBL, Meyerhofstrasse 1, 69117 Heidelberg, Germany; Department of Bioinformatics, Biocenter, University of Würzburg, Würzburg, Germany

## Abstract

The Interactive Tree Of Life (https://itol.embl.de) is an online tool for the management, display, annotation and manipulation of phylogenetic and other trees. It is freely available and open to everyone. iTOL version 6 introduces a modernized and completely rewritten user interface, together with numerous new features. A new dataset type has been introduced (colored/labeled ranges), greatly upgrading the functionality of the previous simple colored range annotation function. Additional annotation options have been implemented for several existing dataset types. Dataset template files now support simple assignment of annotations to multiple tree nodes through substring matching, including full regular expression support. Node metadata handling has been greatly extended with novel display and exporting options, and it can now be edited interactively or bulk updated through annotation files. Tree labels can be displayed using multiple simultaneous font styles, with precise positioning, sizing and styling of each individual label part. Various bulk label editing functions have been implemented, simplifying large scale changes of all tree node labels. iTOL’s automatic taxonomy assignment functions now support trees based on the Genome Taxonomy Database (GTDB), in addition to the NCBI taxonomy. The functionality of the optional user account pages has been expanded, simplifying the management, navigation and sharing of projects and trees. iTOL currently handles more than one and a half million trees from >130 000 individual user accounts.

## Graphical Abstract

**Graphical Abstract:**
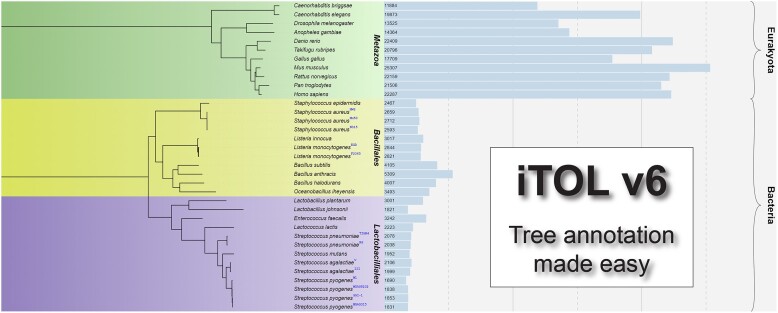

## Introduction

Phylogenetics and phylogenetic trees play pivotal roles in biological and scientific studies, serving as foundational tools for understanding evolutionary relationships and biodiversity.

Numerous tree visualization tools have been developed through the years (e.g. (1–4), including iTOL (5), which was one of the first to introduce tree annotation through diverse supplementary data. Alongside iTOL, many software packages and libraries have emerged, such as the ETE toolkit (6), ggtree (7), tvBOT (8), Evolview (9) or PhyD3 (10), which offer sophisticated tree annotation capabilities.

As the heterogeneity and complexity of data and supplementary data increases, there's a pressing need for tools to adapt and evolve. With version 6, iTOL has undergone a significant redesign, with expanded and streamlined functionality. This redesign aims to simplify data handling and enhance user experience, aligning with the evolving demands of phylogenetic analysis. Here we provide an overview of iTOL’s current features and recent enhancements, emphasizing its continued relevance and significance in scientific research.

### Basic features

**Figure 1.**
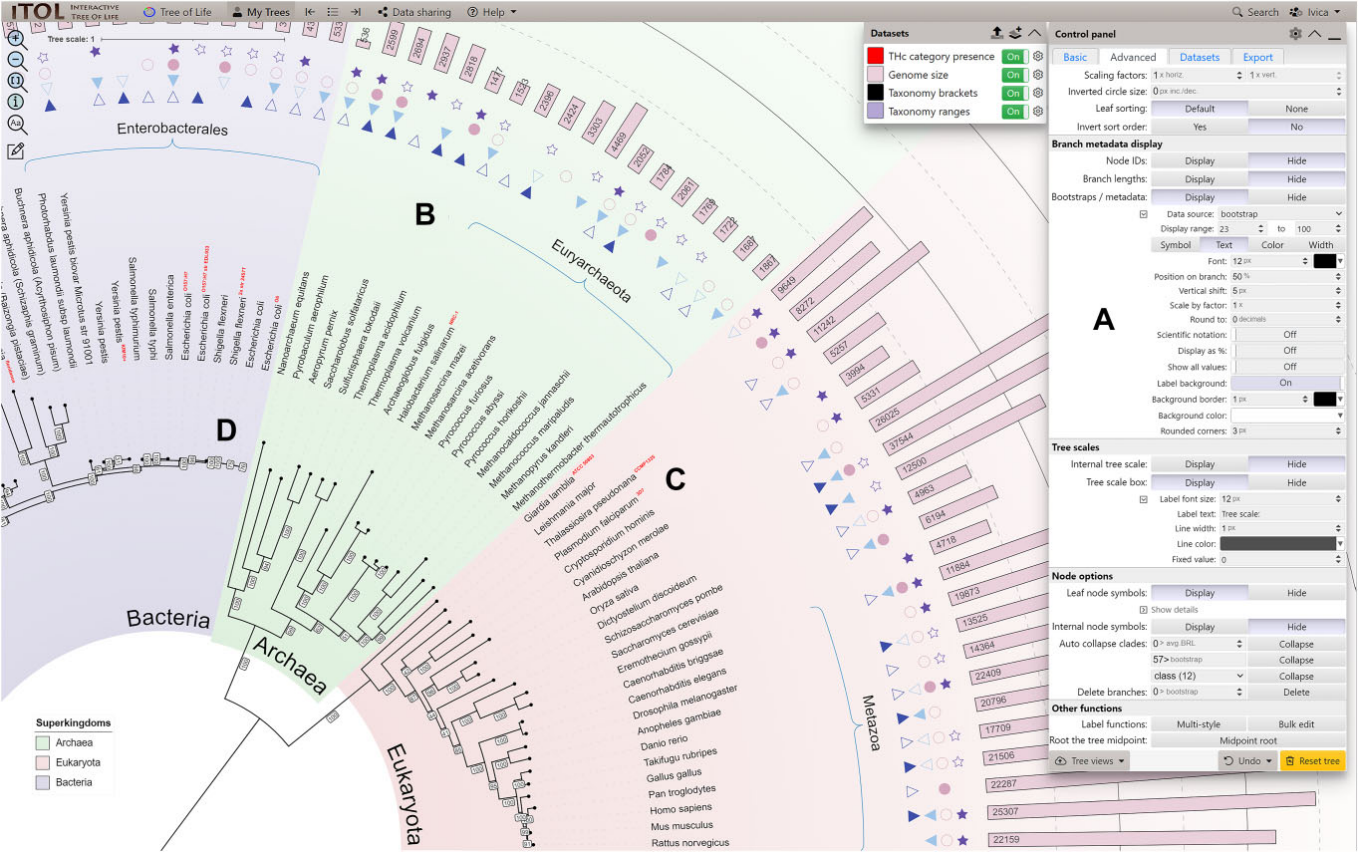
iTOL’s user interface. A phylogenetic tree annotated with several datasets is displayed, highlighting several new features. (**A**) The main control panel has been streamlined, and allows quick access to a larger set of parameters and functions. (**B**) The new ‘Colored/labeled ranges’ dataset type can be visualized as standard color filled shapes, or as various types of brackets. Each range can be separately labeled, with precise positioning of the label relative to the range shape. Gradient color fills and individual border styles can be defined for each range. (**C**) Multiple font styles can be mixed within the tree labels. Styles can be simply defined and applied interactively through the new label style creator. (**D**) Metadata and other branch labels can have custom backgrounds and their position can be fine-tuned relative to the branch. Leaves and internal tree nodes can be marked with custom shapes with user defined colors and borders.

iTOL is an online tool, accessible with any modern web browser (Figure 1). The tree display engine is implemented in pure Javascript, utilizing the HTML5 Canvas element for visualization. The majority of display computations and features are executed by the user's web browser, allowing fine grained interactive control over various display parameters with immediate visualization updates.

### User interface updates

In iTOL version 6, the complete user interface has been rewritten from scratch, utilizing current web technologies and introducing many new convenience functions (Figure 1).

The main control panel has been streamlined and compacted, with collapsible subpanels allowing simple access to a larger set of parameters and functions. Most options contain inline help popups, which can be useful to new users.

iTOL’s help pages have been updated as well, with detailed explanation of all novel features. Most of the functionality is also covered in a set of narrated video tutorials, with crosslinks from the help pages directly to the relevant sections in the videos.

### Input types and basic functions

iTOL supports most commonly used phylogenetic tree formats such as Newick, Nexus (11) and phyloXML (12). Phylogenetic placement files created by EPA (13) and pplacer (14), as well as QIIME 2 trees and annotation files (15) are also supported. With iTOL v6, certain tree branch annotations embedded in the phyloXML files (color and width) will be automatically parsed and applied to the uploaded tree.

All additional data used for various types of tree annotation are provided in plain text files, and visualized by simply dragging and dropping them onto the user's web browser. Annotation files now support simple assignment of annotations to multiple tree nodes through substring matching, including full regular expression support, making them more compact and lowering the amount of work needed.

iTOL provides most common functions available in any phylogenetic tree viewer. Various tree display formats are supported: phylograms or cladograms, rooted or unrooted, rectangular or radial.

iTOL can manipulate the trees in various ways, and basic editing functions allow users to interactively delete or move single nodes or whole clades. Clades can also be pruned or collapsed, either manually or automatically, based on various parameters (such as associated bootstrap values, average branch length distances or any other numeric metadata value associated with tree nodes). iTOL v6 introduces support for the collapsing of clades through a simple text file with a list of nodes to collapse. Such files can be manually created, or exported from trees with interactively collapsed clades, allowing simple transfer of the collapsed clade status to other user trees.

Trees can be re-rooted manually on any node, or automatically using the midpoint rooting method. Tree leaves can be sorted in various ways, either manually or automatically, and their order rearranged in each individual clade.

Performing large scale editing of tree node labels in iTOL has been simplified by the introduction of the bulk label editor. It provides several convenience functions which can be applied to all tree labels with a single button click, from simple removal of quotes to powerful regular expression text replacements.

### Automatic taxonomy assignments

iTOL’s automatic taxonomy assignment functionality provides a simple way of deducing the correct labels and classes of all tree nodes based on external taxonomy databases. In addition to the NCBI taxonomy database (16), iTOL now also supports GTDB (Genome Taxonomy Database) (17). The taxonomy assignment function requires trees with taxonomy IDs (NCBI or GTDB) at their leaf nodes. It will replace the ID labels with correct taxonomic names, as well as resolve the correct names and classes of all internal tree nodes, based on their child nodes.

A complete overview of changes and functions added since the last publication (18) are listed on the iTOL’s version history page (https://itol.embl.de/version_history.cgi).

### Tree annotation

iTOL v6 introduces various new annotation features, extended functionality in the visualization of existing dataset types, as well as a new dataset type, colored/labeled ranges (Figure 1). Limited colored leaf ranges were available in iTOL as a separate annotation feature since the initial release. The new dataset type replicates and greatly extends this functionality with many new features. Since colored ranges are now an independent dataset, there can be multiple different versions present on a single tree, allowing users to simply highlight different parts of the tree as required. Ranges can now be filled with color gradients, and highlighted with individually styled borders of varying widths and colors. Each colored range can be labeled separately, with automatic positioning of the label which can be fine-tuned by the user. Colored ranges can automatically cover not only the tree structure itself, but also any external datasets displayed outside the tree, allowing users to easily highlight the relevant sections of the complete annotated tree. In addition to the standard colored shapes, ranges can be visualized as various types of brackets, further extending their possible uses.

### Extended support for node metadata

iTOL v6 expands the node metadata handling and visualization options with several new features. In addition to automatic import of bootstrap values and metadata from MRBAYES (19) and The New Hampshire X (NHX) formatted trees, custom metadata values can now be imported or updated directly from user generated plain text annotation files. In addition to the bulk importing or editing, an interactive metadata editor has been implemented, allowing simple access to each individual node's metadata values. The metadata editor can also be used to define node classes, which can be used in the automatic clade collapsing function.

During metadata visualization on trees where multiple metadata values are available per node, the display can now be filtered via individual thresholds for each metadata category with full support for both ‘AND’ and ‘OR’ Boolean operators.

### Tree labels with multiple font styles

The ability to mix different font styles within tree labels is a feature that was often requested by users, and is now available in iTOL v6. Through a simple interactive form directly in the web interface, users can split the labels into different parts as desired, and define their individual font styles. For each part of the label, it is possible to define a separate color, size, font family, bold/italic style and sub or superscript position. Additionally, the position of each part can be fine-tuned vertically and horizontally as needed.

Several filtering options allow the application of styles to a subset of labels only, for example by including or excluding labels which match a particular string, or by requiring a specific number of parts to be present.

### User accounts and tree management

**Figure 2.**
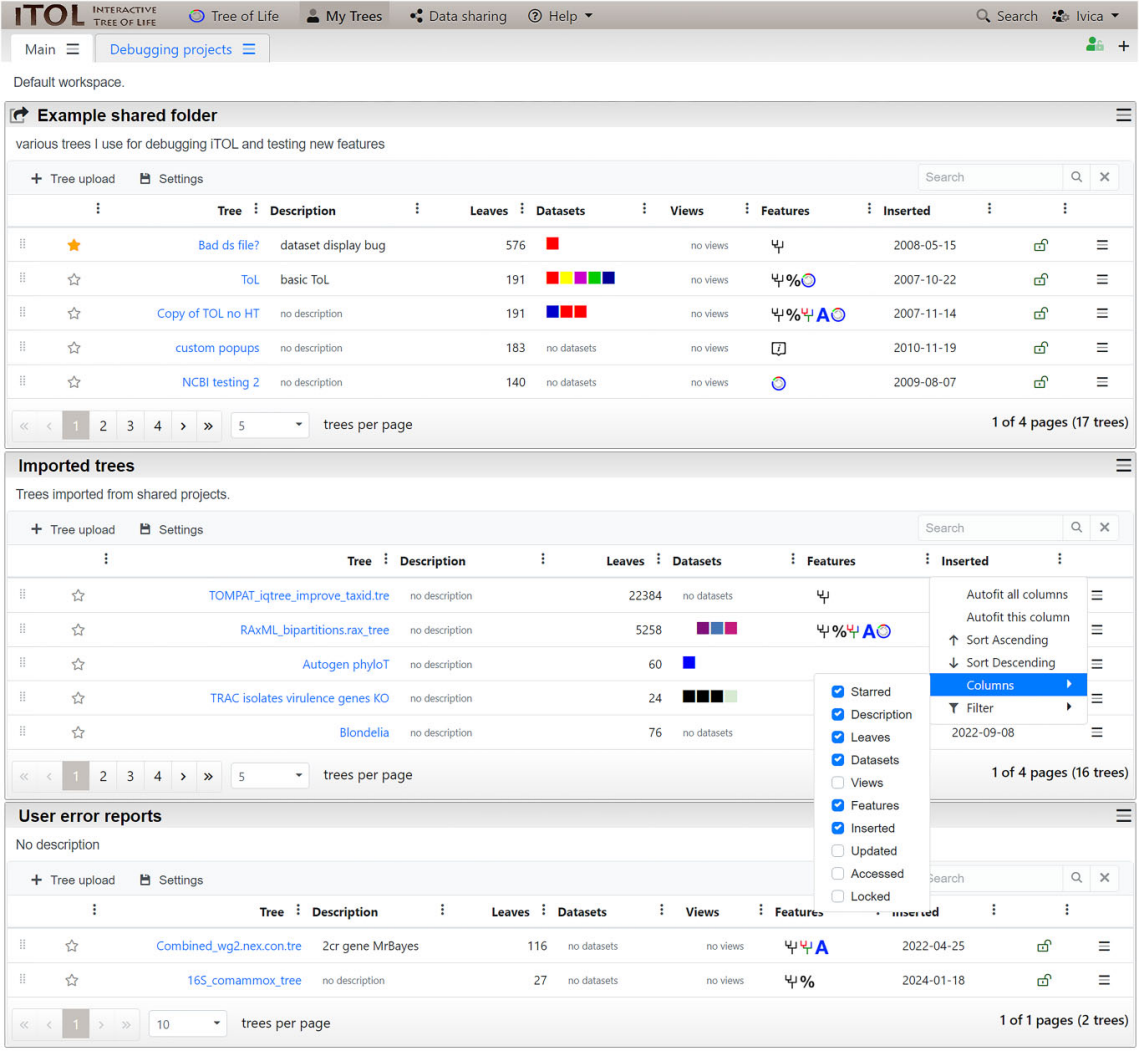
Illustration of the user account page redesign in iTOL v6. Trees can be organized into various workspaces and projects. Projects can be collapsed and rearranged, or moved between workspaces. Tree lists in each project can be customized according to user preferences, where columns can be sorted, reordered or hidden. Trees can be rearranged or moved to other projects by direct drag and drop. All settings can be saved individually for each project, or applied globally.

In addition to the anonymous direct tree upload and annotation, iTOL provides a personal account system, which currently has >130 000 registered users, managing more than 1 600 000 uploaded trees. The current version brings a complete redesign of the user account pages (Figure 2). Users can organize their trees into various workspaces and projects. Trees uploaded into each project are displayed in a table with several advanced features. Tree lists can be sorted on any of the available columns, while the columns can be toggled and reordered as desired. For projects with many trees, users can specify the paging size of the table, making the navigation through the workspace simpler. All these settings can be applied to each project separately, or defined as user defaults for all their workspaces and projects.

Several new tree attributes have been introduced, like the ability to mark important trees with a star, or to prevent any public access to a particular tree.

In addition to the standard user accounts created directly, iTOL now supports Single Sign-On Authentication (SSO) via Google or Microsoft services for both login and registration. Users can simply use their existing external accounts in iTOL, without the need to remember new passwords or provide any additional information.

### Export

One of iTOL’s primary uses is the creation of high-quality figures for publication or inclusion into other documents. Due to constantly increasing number of active users, the backend server has been extended to make the tree export faster. To prevent the overloading of the export server in peak usage times, we developed a simple queueing system, which processes user exports sequentially. Introduction of the export queuing system made the process more stable for all users, and increased the overall response times of the server.

Taken together, iTOL version 6 provides numerous improvements of both backend and frontend user interfaces, introduces many new annotation features and streamlines the tree annotation process, improving the user experience.

## Data Availability

iTOL is free and open to all users without login requirement at https://itol.embl.de/.
